# Difluoroboron β-diketonate polylactic acid oxygen nanosensors for intracellular neuronal imaging

**DOI:** 10.1038/s41598-020-80172-w

**Published:** 2021-01-13

**Authors:** Meng Zhuang, Suchitra Joshi, Huayu Sun, Tamal Batabyal, Cassandra L. Fraser, Jaideep Kapur

**Affiliations:** 1 Department of Chemistry, University of Virginia, Charlottesville, VA 22904 USA; 2 Department of Neurology, University of Virginia, Charlottesville, VA 22903 USA; 3 Department of Neuroscience, University of Virginia, Charlottesville, VA 22903 USA; 4 UVA Brain Institute, University of Virginia, Charlottesville, VA 22903 USA

**Keywords:** Neuroscience, Chemistry

## Abstract

Critical for metabolism, oxygen plays an essential role in maintaining the structure and function of neurons. Oxygen sensing is important in common neurological disorders such as strokes, seizures, or neonatal hypoxic–ischemic injuries, which result from an imbalance between metabolic demand and oxygen supply. Phosphorescence quenching by oxygen provides a non-invasive optical method to measure oxygen levels within cells and tissues. Difluoroboron β-diketonates are a family of luminophores with high quantum yields and tunable fluorescence and phosphorescence when embedded in certain rigid matrices such as poly (lactic acid) (PLA). Boron nanoparticles (BNPs) can be fabricated from dye-PLA materials for oxygen mapping in a variety of biological milieu. These dual-emissive nanoparticles have oxygen-insensitive fluorescence, oxygen-sensitive phosphorescence, and rigid matrix all in one, enabling real-time ratiometric oxygen sensing at micron-level spatial and millisecond-level temporal resolution. In this study, BNPs are applied in mouse brain slices to investigate oxygen distributions and neuronal activity. The optical properties and physical stability of BNPs in a biologically relevant buffer were stable. Primary neuronal cultures were labeled by BNPs and the mitochondria membrane probe MitoTracker Red FM. BNPs were taken up by neuronal cell bodies, at dendrites, and at synapses, and the localization of BNPs was consistent with that of MitoTracker Red FM. The brain slices were stained with the BNPs, and the BNPs did not significantly affect the electrophysiological properties of neurons. Oxygen maps were generated in living brain slices where oxygen is found to be mostly consumed by mitochondria near synapses. Finally, the BNPs exhibited excellent response when the conditions varied from normoxic to hypoxic and when the neuronal activity was increased by increasing K^+^ concentration. This work demonstrates the capability of BNPs as a non-invasive tool in oxygen sensing and could provide fundamental insight into neuronal mechanisms and excitability research.

## Introduction

Molecular oxygen (O_2_) plays an essential role in living systems^1,2^. In eukaryotic cells, oxygen is required as a terminal electron acceptor in the aerobic metabolic process, known as oxidative phosphorylation^3^. This reaction takes place in mitochondria, during which the electrons transfer from an electron donor to an electron acceptor or oxygen, and energy released from electron flow is used to transform adenosine diphosphate (ADP) to adenosine triphosphate (ATP). The ATP generated during phosphorylation drives many cellular activities and processes and maintains biochemical function.

Molecular O_2_ is essential in the structural and functional integrity of the brain. Though the brain is only 2% of the body’s mass, it consumes nearly 20% of O_2_ in the resting body^4^. Neuronal computation is energetically expensive, which requires an adequate supply of ATP or O_2_ for activities ranging from maintaining electrochemical gradients to releasing and recycling synaptic vesicles^5,6^. Hypoxia–ischemia is a major cause of brain injury in humans, including conditions such as hypoxic–ischemic encephalopathy^7^, stroke^8,9^, seizures^8,10,11^, periventricular leukomalacia^12^, and even Alzheimer’s disease^13,14^. Therefore, it is important to understand neuronal oxygen levels in various neurological disorders.

Oxygen sensing with good spatial and temporal resolution can offer new fundamental insight into brain function. Current methods to assess O_2_ levels in biomedicine include polarographic oxygen electrodes (also known as Clark electrodes), radioisotope techniques (positron emission tomography and single-photon emission computed tomography), magnetic resonance techniques (e.g., fMRI), and optical methods based on hemoglobin O_2_ saturation and phosphorescence quenching^15^. Some of these methods have significant limitations of equipment cost, invasiveness, or low spatial resolution. For example, polarographic electrodes are invasive and limited to single-point measurements; radioisotope techniques only show regions of hypoxia. Also, hemoglobin-based detection is indirect and only provides an estimation of O_2_ levels in the blood; and fMRI assessments are restricted to long time scales.

Oxygen nanosensors based on phosphorescence quenching can address some of these limitations of spatial and temporal resolution with improved micron-level spatial and sub-seconds temporal resolution^16–20^. Efforts have been made during the past decades in applying phosphorescence probes as oxygen sensors in neuronal tissue^21–23^. For example, Ingram et al. developed platinum (II) octaethylporphine ketone (PtOEPK) and nanocrystals quantum dot (NQD) blends to measure the interstitial oxygen concentration of active brain slices through fluorescence resonance energy transfer (FRET)^24^. The Papkovsky group designed cell-permeable probes based on a phosphorescent Pt(II)-tetrakis(pentafluorophenyl)porphyrin (PtPFPP) dye and a fluorescent poly(9,9-diheptylfluorene-alt-9,9-di-p-tolyl-9H-fluorene standard^25^. These methods rely on blending a phosphorescence probe, a fluorescence standard, and a polymer matrix altogether, rendering the synthetic and preparation process somewhat complex. In addition, currently, there are few reports measuring intraneuronal oxygen levels.

Difluoronboron β-diketonates are a class of compounds with excellent luminescence properties, including large extinction coefficients, high quantum yields, and two-photon absorption. Their emission is easily tunable by the chemical structure to span across the visible range, making them useful as bioimaging reagents. When a boron dye is confined in a rigid matrix such as poly (lactic acid) (PLA), the fluorescence is retained, and at room temperature, the phosphorescence is greatly enhanced^26^. This excellent feature offers an opportunity for boron dye polymers to serve as ratiometric oxygen sensors, where the oxygen-insensitive fluorescence is the internal standard for self-calibration, and the quenchable oxygen phosphorescence senses response on local oxygen level changes. With the presence of biocompatible and biodegradable PLA, the boron dye polymers can be fabricated in multiple ways for biological applications, including nanoparticles, thin-film, and nanofibers^27^. As the fluorescence standard, the phosphorescence sensor and the matrix are all combined in a single macromolecular material, boron dye polymers show unique advantages over many other phosphorescent probes. First, the dye preparation and fabrication process are cost-effective and facile within a few steps. Second, in contrast to the boron system, for ratiometric oxygen sensing, where the standard and the sensor are two separate compounds, it can be challenging to have the same photobleaching rate for both dyes in quantitative analysis. Also, both the boron dye and the polymer can be easily adapted for commonly used imaging setups and multiple biological purposes. In this work, the oxygen sensing capability of iodine substituted difluoroboron β-dibenzoylmethane PLA (BF_2_dbm(I)PLA) nanoparticles was employed to study brain activity. In particular, the brain slices were labeled by the cell-penetrating nanoparticles, and ratiometric oxygen maps were generated. It was found that the neuronal cell bodies consume less oxygen than dendrites and synapses, where mitochondria are more concentrated.

## Results and discussions

### Nanoparticles

**Figure 1:**
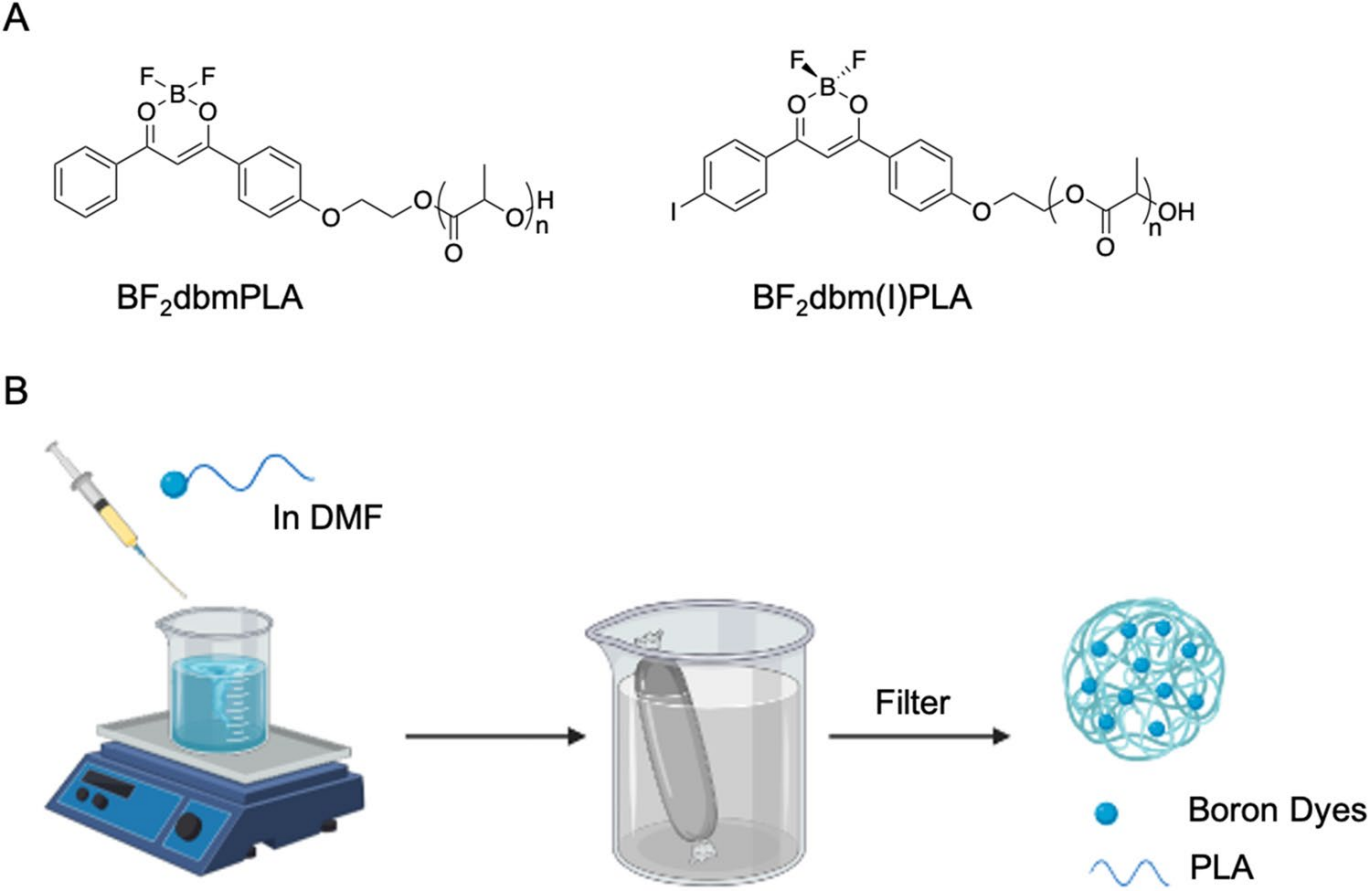
Nanoparticle composition and fabrication. (**A**) Chemical structures of blue-emitting BF_2_dbmPLA and dual-emissive, oxygen sensing BF_2_dbm(I)PLA. (**B**) Nanoparticles are fabricated by the nanoprecipitation method.

The boron dye polymers were synthesized as previously described^26^. In general, the boron dye with a primary alcohol initiator group was dissolved in melted lactide. The polymers were grown in the presence of tin catalyst via ring-opening polymerization. The crude polymers were purified by dissolving in a minimal amount of CH_2_Cl_2_, followed by addition into cold methanol to remove residual small organic dyes and lactide. The polymers were characterized by NMR and GPC. The nanoparticles were fabricated by a method known as nanoprecipitation, which involves interfacial deposition due to displacement of solvent (i.e., DMF) with non-solvent (i.e., DI water). In this work, two types of nanoparticles were fabricated and characterized as illustrated in Fig. 1. Blue nanoparticles BF_2_dbmPLA (blue NPs) were chosen for labeling primary neuronal cells because their high quantum yield and bright fluorescence make them easily detected and ideal for cell tracking^27^. Oxygen sensing nanoparticles BF_2_dbm(I)PLA (O_2_ NPs) were used to generate ratiometric oxygenation maps where the fluorescence (λ_F_ = 450 nm) acts as an internal standard and the oxygen-sensitive phosphorescence (λ_P_ = 535 nm) is the sensor. Nanoparticle characteristics have been extensively studied in previous reports, as listed in Table 1. The maximum UV excitation wavelengths for both nanoparticles (blue NPs: λ_ex_ = 396 nm; O_2_ NPs: λ_ex_ = 405 nm) are compatible with 405 nm laser excitation.

**Table 1:** Nanoparticle characteristics.

Nanoparticles	Optical properties					Physical properties		
	λ_ex_^a^ (nm)	λ_F_^b^ (nm)	λ_P_^c^ (nm)	τ_F_^d^ (ns)	τ_P_^e^ (ms)	R_*h*_^f^ (nm)	PD^f^ (%)	ζ^g^ (mV)
BF_2_dbmPLA	396	439	509	3.84	200	52	15	− 28
BF_2_dbm(I)PLA	405	450	535	0.54	4.37	71	24	− 24

^a^Absorption maxima. ^b^Fluorescence emission maxima excited at 369ˆnm. ^c^Phosphorescence maxima in the delayed emission spectrum excited at 369 nm with a 0.5 ms delay. ^d^Fluorescence lifetime monitored at the emission maximum. ^e^Phosphorescence lifetime monitored at the phosphorescence maximum. ^f^Hydrodynamic radius and polydispersity (PD) determined by dynamic light scattering. ^g^Zeta potential measured by ZetaSizer.

The physical properties, including radius and zeta potential, were measured by DLS and ZetaSizer. It is believed that size and surface charge are critical factors affecting the interaction between nanomaterials and biological components, consequently the efficiency of localization into cells and tissue. As listed in Table 1, the hydrodynamic radius for blue NPs was 52 nm with a polydispersity of 15%, and for O_2_ NPs, it was 71 nm with a polydispersity of 24%, both of which are typical values for the family of BF_2_bdkPLA and are consistent with previous results^28,29^. The data suggested the polymers successfully self-assemble into nanoparticles. In addition, nanoparticles with radii ranging from 20 to 200 nm can be readily internalized via endocytosis; they are large enough to avoid renal clearance and small enough for clearance from the spleen^30^. Based on zeta potential, blue NPs and O_2_ NPs were negatively charged, in accordance with commonly developed PLA nanoparticles. An early report from Dante et al. revealed that NPs with negative charge rapidly localize on neuronal membranes, while those with positive or neutral charge showed no or slow interaction with neurons^31^. With the size and zeta potential suitable for endocytosis, we hypothesized that the boron dye polymer NPs would be internalized by neurons and respond to intracellular oxygen variations.

### Cellular uptake

**Figure 2:**
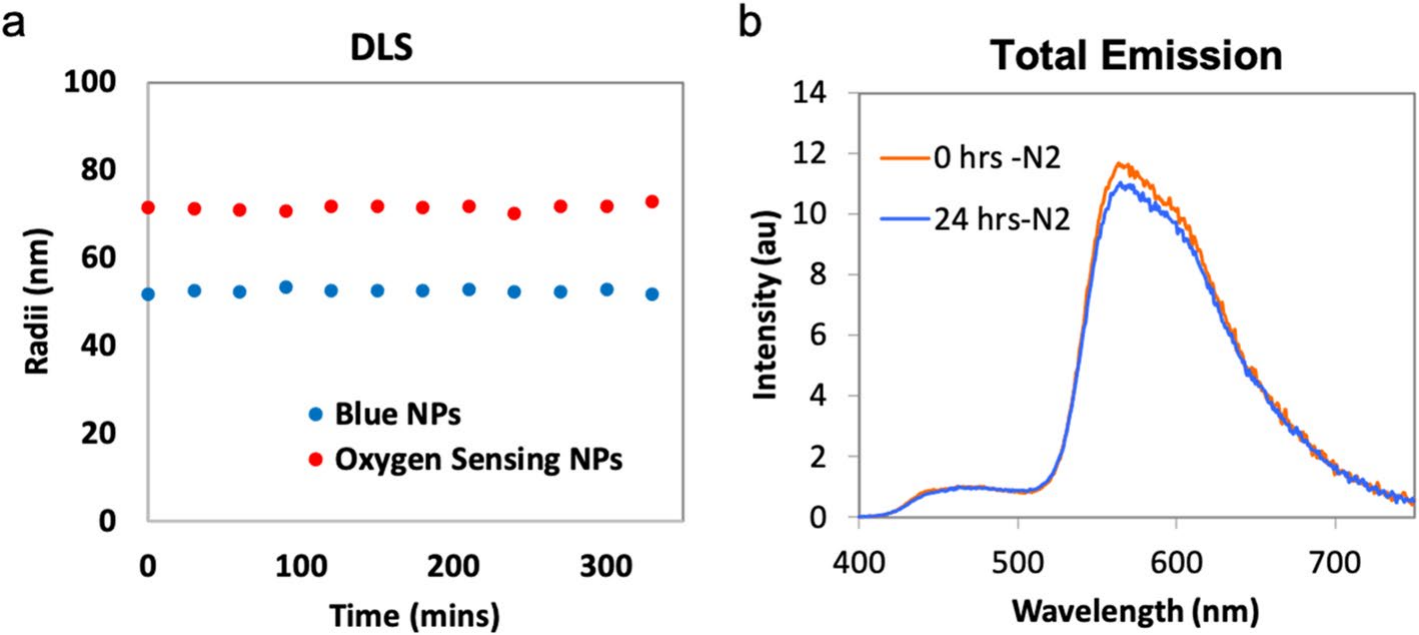
The stability of NPs. (**a**) DLS traces for NPs. The radii were recorded every 30 min. (**b**) The stable oxygen-sensing ability of NPs was confirmed by measuring the emission before and after incubation at 37 °C for 24 h.

To confirm the intracellular uptake, the stability (i.e., no aggregation) of NPs was firstly evaluated in a neuronal medium at 37 °C over time as nanoparticles fabricated from pure hydrophobic polyesters are prone to precipitate in an aqueous solution. The NPs were diluted with neuronal medium (included in the methods) at a final concentration of 500 μg/mL, monitored by DLS every 30 min over 6 h, a period of time sufficient for incubation and imaging. It was observed that the NPs could maintain their size without aggregation for the duration (Fig. 2a). We also evaluated the optical stability of O_2_-sensing NPs. The emission of O_2_ NPs suspended in ACSF solution was measured before and after incubation at 37 °C for 24 h. The two spectra were similar, indicating that these particles' phosphorescence O_2_-sensing ability was unaffected over the incubation period (Fig. 2b).

**Figure 3:**
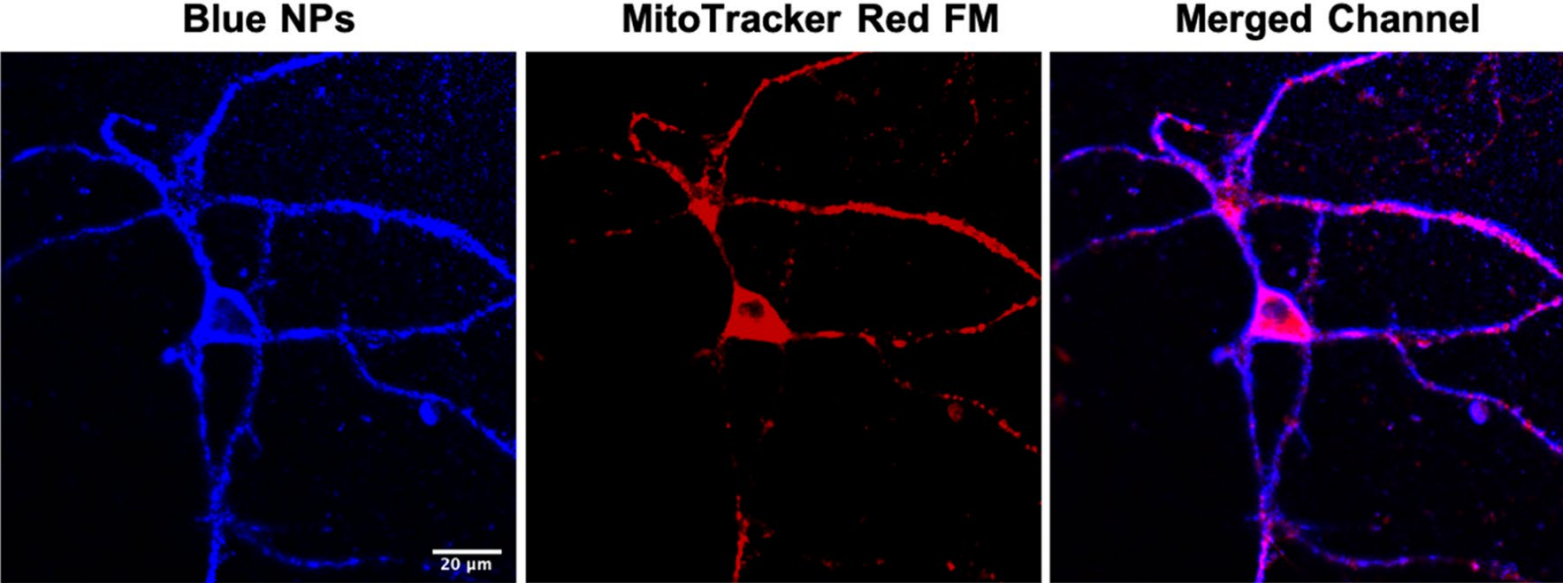
Neuronal imaging under confocal microscopy. The images showed labelling from blue NPs (left) and MitoTracker Red (middle) and merged channel from blue and red (right). The excitation wavelength is 405 and 595 nm.

The blue NPs suspended in the neuronal medium were then used to label primary neuronal cultures due to their high quantum yield (Φ_F_ = 0.99), ideal for tracking cellular internalization^27,28^. The cultures were 14 DIV at the time of the experiment, with well-formed neuronal structures, including synapses. The mitochondria were labeled with MitoTracker Red FM, the maxima fluorescence of which (λ_F_ = 644 nm) is far away from the blue NP emission. With confocal microscopy, we found that blue NPs showed non-specific and uniform interactions and distribution with living cells (e.g., neurons and glial cells). Neuronal cell bodies (i.e., a perinuclear region of cytoplasm), dendrites, and synapses can be easily visualized with the bright blue fluorescence (Fig. 3; left). To further ensure that the NPs enter the neurons and can measure intracellular oxygen level, a red mitochondria dye, MitoTracker Red FM, with emission having no interference with NPs fluorescence, was selected to label neurons (Fig. 3; middle). By specifically binding to the mitochondrial membrane, the MitoTracker targets mitochondria where the oxygen is consumed to generate ATP and energy for a variety of neuronal activities. As shown in Fig. 3, the blue channel of NPs emission and red channel of MitoTracker Red FM emissions were matched, revealing the colocalization of these two dyes. The colocalization of these two dyes suggested that effective intracellular oxygenation can be detected from the oxygen-sensing NPs.

### Slice imaging and toxicity

**Figure 4:**
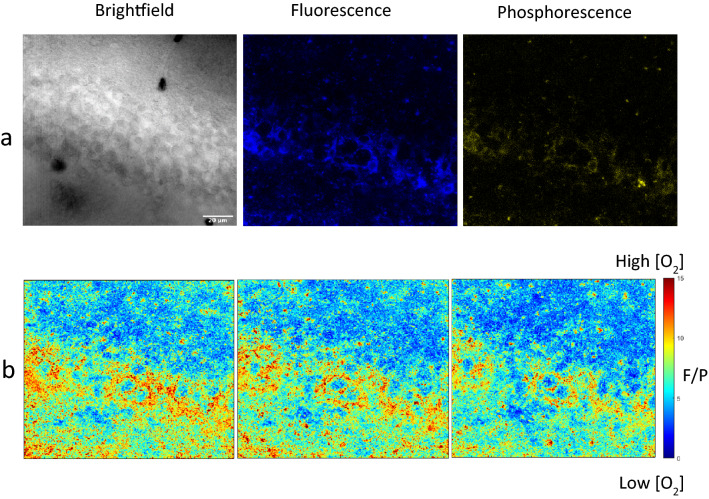
Brain slice imaging with O_2_ NPs. (**a**) Brightfield, fluorescence, and phosphorescence emission of the hippocampus DGC layer. (**b**) Ratiometric imaging when the oxygen supply is off over time.

Dual-emissive O_2_ sensing NPs possess oxygen-insensitive fluorescence and room temperature oxygen-sensitive phosphorescence. As oxygen concentration increases, the phosphorescence intensity decreases, whereas the fluorescence remains unchanging (Figure S1), serving as useful agents for ratiometric oxygen sensing^29,32,33^. At a range of 0–21% O_2_, the O_2_ NPs had a linear relationship between the fluorescence to phosphorescence (F/P) ratio and oxygen concentration. After the cellular uptake studies in cultured neurons, O_2_ sensing NPs were tested using living brain slices. The brain slices were incubated with a solution containing 500 μg/mL O_2_ NPs, during which they were oxygenated with 95% O_2_. The dentate gyrus within the hippocampus was imaged because the neuronal circuits activated during seizures involve the hippocampus, in particular, the dentate granule cells (DGC), which regulate the flow of signal within the neuronal network. It is thus, of interest and importance to investigate how the oxygen level varies in the DGC layer. During imaging, the brain slice was sealed in a closed chamber with a circulation solution containing 95% O_2_. The results were based on the analysis of a group of 10 slices from seven mice. As shown in Fig. 4a, the DGC layer is visible in brightfield, and the emission captured from fluorescence was indicative of the slice stained by nanoparticles. It was observed that the DGC layer has brighter fluorescence. This may be due to the great number of cells tightly packed here, resulting in more nanoparticles loaded. The phosphorescence was detected simultaneously along with fluorescence using a separate emission filter range (Fig. 4a).

The fluorescence and phosphorescence were analyzed to generate ratiometric images with an F/P ratio correlating with oxygen levels (Fig. 4b and Figure S2). By taking the ratio of F/P, the uneven distribution of nanoparticles within the slice can be self-calibrated by the oxygen-insensitive fluorescence (i.e., internal standard). While the brain slice is supplied with oxygen, a subtle contrast between dendrites and cell bodies was observed; the oxygen level in dendrites is lower than in cell bodies of granule layers. The oxygen supply was stopped to test the NP response to oxygen changes in tissue, and imaging was taken every 5 min for 10 min (i.e., data points at 0, 5, 10 min). The F/P ratio generally decreased over time (i.e., oxygen levels decreased), and the contrast between dendrites and cell bodies became more pronounced. Oxygen is consumed by mitochondria in neuronal cell bodies, at dendrites, and at synapses. At dendrites where synapses are located, there are many neuronal activities taking place; therefore, mitochondria are more concentrated there compared to cell bodies. The mitochondria rich synapses consume more oxygen and consume it more quickly, leading to lower oxygen levels.

The F/P ratio was determined in a region of interest encompassing dentate granule cells and their processes. The F/P ratios in 12 slices at 5 min and 10 min after oxygen supply to the slices was stopped shows increased phosphorescence at 5 min and 10 min with respect to the F/P ratio at baseline (Table 2).

**Table 2:** Baseline (0 min)-normalized F/P values per unit area below a threshold nanoparticle characteristics.

	Slice 2	Slice 3	Slice 4	Slice 5	Slice 6	Slice 7	Slice 8	Slice 9	Slice 10	Slice 11	Slice 12	Median	SD
0 min	1.0	1.0	1.0	1.0	1.0	1.0	1.0	1.0	1.0	1.0	1.0	1.0	0
5 min	1.448	1.207	1.033	1.742	0.903	1.443	1.625	1.449	1.882	1.098	0.903	1.443	0.335
10 min	2.029	1.685	1.064	2.304	0.887	1.084	1.478	1.478	2.470	1.241	1.115	1.478	0.533

The threshold was kept constant for all the slices. Null hypothesis H0: No observable difference of baseline normalized F/P per unit area between a pair of time instants. The normalized F/P at 0 min is non-normal, whereas KS test fails to reject that the normalized F/P values are sampled from a Normal distribution in cases of 5 min (*P* = 0.86) and 10 min (*P* = 0.83). H0 is rejected when normalized F/P values are compared at 0 min and 5 min (*P* = 0.0077, Wilcoxon rank-sum test) and at (0 min, 10 min) with *P* = 0.0005 (Wilcoxon rank-sum test). Pairwise T-test fails to reject H0 for (5 min, 10 min).

**Figure 5:**
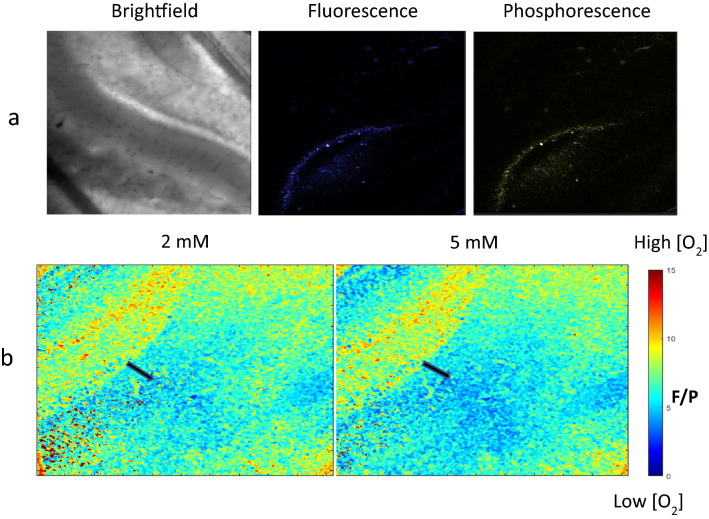
Potassium concentration effect on neuronal activity. (**a**) Ratiometric images under low K^+^ (2 mM) and high K^+^ (5 mM) concentration. (**b**) Ratiometric imaging in the presence of 2 mM and 5 mM extracellular KCl. Black arrows mark the regions with lower oxygen.

To further support the statement that oxygen is consumed at the site of synaptic activity, the potassium concentration in perfusion solution was increased from 2 to 5 mM. Potassium regulates neuronal activity, and an elevated extracellular K^+^ level is known to affect synaptic potentials and increase neuronal activities^34,35^. We performed slice oxygen imaging under different potassium concentrations. When potassium concentration was increased, the regions away from the molecular layer were more hypoxic as suggested by the F/P ratio in the ratiometric oxygen imaging in Fig. 5a,b and Table 3.

**Table 3:** F/P per unit area below a threshold normalized to the 2 mM measurement.

	Slice 1	Slice 2	Slice 3	Slice 4	Slice 5	Slice 6	Median	SD
2 mM	1.0	1.0	1.0	1.0	1.0	1.0	1.0	0
5 mM	2.029	1.121	1.080	1.203	1.107	1.035	1.114	0.3796

Null hypothesis H0: No observable difference of baseline normalized F/P per unit area between slices maintained in a solution containing 2 mM and a 5 mM K^+^. The normalized F/P at 0 min is non-normal, whereas KS test fails to reject that the normalized F/P values are sampled from a Normal distribution in cases of 2 mM solution (*P* = 0.23). Wilcoxon rank-sum test rejects H0 (*P* = 0.022).

The results from brain slice imaging demonstrated the effectiveness of oxygen sensing nanoparticles in studying brain activity by measuring intracellular oxygen levels.

These proof of principle studies performed using cultured neurons or acutely isolated brain slices revealed that the NPs could be effectively used to obtain insights into tissue oxygen levels. The efficacy of NPs in vivo remains to be determined. Evaluation of changes in tissue oxygenation in deeper brain structures may also be challenging and the effect of scattering and absorbance by of the fluorescence and phosphorescence signal will have to be resolved, likely through further modifications of the NPs.

**Figure 6:**
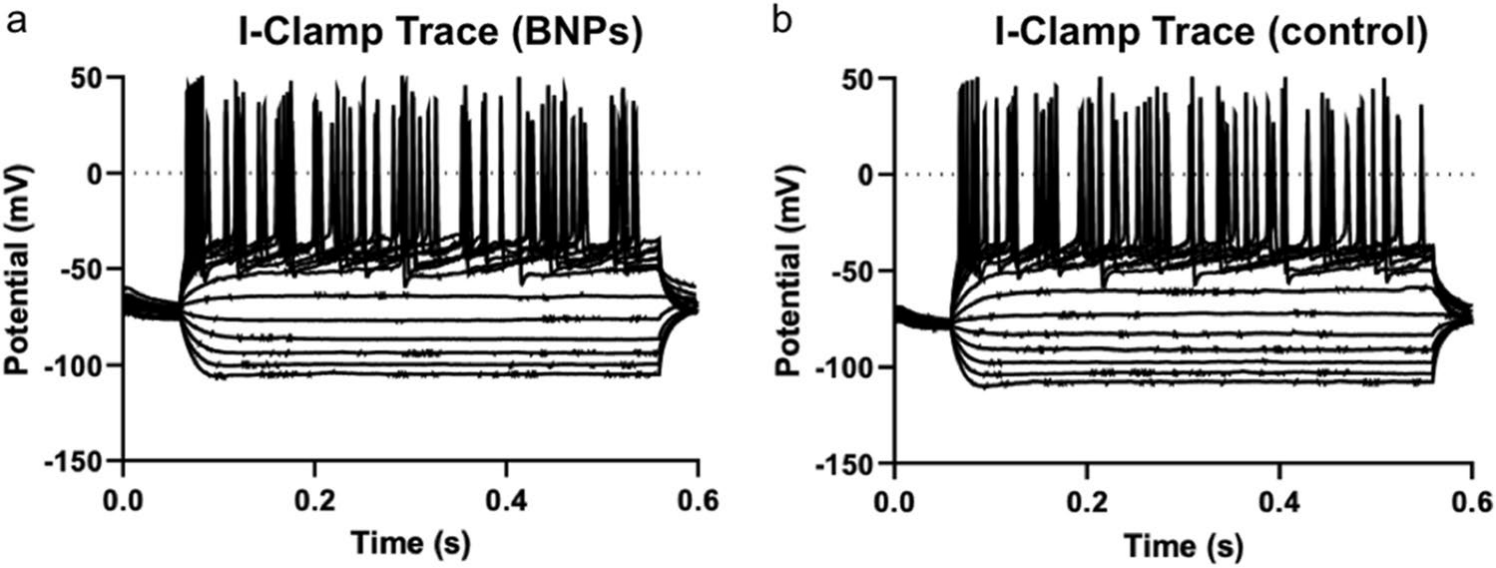
I-Clamp trace of neurons in the oxygen sensing nanoparticle treated slice (**a**) and the control slice (**b**).

For biomedical imaging, it is essential to verify that the imaging agent has no toxic effect on biological systems. The neurons in the DGC layer were recorded by a whole-cell patch-clamp to investigate the electrophysiological properties of the brain slice after the application of oxygen sensing nanoparticles. The data were collected in a group of five slices from four mice. As shown in Fig. 6 and Figure S3, the nanoparticle treated slice showed a nearly identical I-clamp trace with the control. Six membrane properties, including resting membrane potential, membrane resistance, membrane time constant, action potential wide, action potential threshold, action potential amplitude, were collected, all of which showed no significant difference between the nanoparticle treated slice and the control slice. The electrophysiology experiments confirmed that oxygen sensing nanoparticles did not measurably alter the electrophysiological properties of the neurons.

## Conclusions

Oxygen nanosensors based on phosphorescence quenching can provide information about the brain with improved micron-level spatial and sub-seconds temporal resolution. In this work, the boron nanoparticles were used to study brain activity. Specifically, primary neuronal cell cultures were labeled by blue nanoparticles from BF_2_dbmPLA to confirm intracellular uptake. Electrophysiology experiments revealed that the application of nanoparticles did not have a significant effect on the electrophysiological properties of neurons. Oxygen sensing nanoparticles based on BF_2_dbm(I)PLA was able to generate ratiometric oxygen maps for brain slices. In ex vivo brain slices, it was found that the neuronal cell bodies consume less oxygen than dendrites and synapses where mitochondria are more concentrated for synaptic activities. These data suggested the capability of boron nanoparticles serving as powerful and non-invasive sensing agents in the brain. Future work will involve oxygen imaging in the living animal and in combination with seizures induced via a cobalt model to study how oxygen levels are related to seizures.

## Methods

All experiments involving mice were performed according to a protocol approved by the University of Virginia Animal Care and Use Committee (ACUC, protocol number 4093). All methods were carried out in accordance with relevant guidelines and regulations. The study was carried out in compliance with the ARRIVE guidelines. All chemicals were obtained from Sigma Aldrich (St. Louis, MO, USA) without further purification unless otherwise stated.

### Dye-polymer synthesis and characterization

The boron dye-polymer was prepared according to a previously reported method^26^. ^1^H NMR spectra were recorded on a Varian VMRS/600 (600 MHz) instrument in CDCl_3_ unless otherwise indicated. ^1^H NMR resonances were referenced to the residual protiochloroform signal at 7.260 ppm. Coupling constants are given in hertz. Polymer molecular weights (MW) and polydispersity indices (Đ) were determined by gel permeation chromatography (GPC) (THF, 25 °C, 1.0 mL/min) using multi-angle laser light scattering (MALLS) (λ = 658 nm, 25 °C) and refractive index (RI) (λ = 658 nm, 25 °C) detection. Polymer Laboratories 5 µm mixed-C columns (guard column plus two columns) along with Wyatt Technology (Optilab T-rEX interferometric refractometer, miniDAWN TREOS multi-angle static light scattering (MALS) detector, ASTRA 6.0 software) and Agilent Technologies instrumentation (series 1260 HPLC with diode array (DAD) detector, ChemStation) were used in GPC analysis. The incremental refractive index (*dn*/*dc*) was calculated by a single-injection method assuming 100% mass recovery from the columns. UV–vis spectra were recorded on a Hewlett–Packard 8452A diode-array spectrophotometer.

### Luminescence measurements

Steady-state fluorescence spectra for the nanoparticle suspensions were recorded on a Horiba Fluorolog-3 Model FL3-22 spectrofluorometer (double-grating excitation and double-grating emission monochromator) after excitation. Optically dilute aqueous solutions of the nanoparticles, with absorbance < 0.1 au, were prepared in 1 cm path length quartz cuvettes. Fluorescence spectra were obtained under ambient conditions (i.e., air, ~ 21% oxygen in volume). For phosphorescence measurements, vials were capped with a 12 mm PTFE/silicone/PTFE seal (Chromatography Research Supplies) and were continuously purged with analytical grade N_2_ (Praxair) for 5 min before measurements. For 21% O_2_ (i.e. air), measurements were taken under ambient conditions (open vial, no cap). Oxygen calibration was performed as previously described with Cole-Palmer flow gauges with mixtures of analytical grade O_2_, N_2,_ and 1% O_2_ (in N_2_) gases (Praxair)^29^.

### Nanoparticle fabrication

Nanoparticles were fabricated as previously reported^36^. The polymer (~ 3.0 mg) was dissolved in DMF (3 mL) and was added dropwise to rapidly stirred DI water (27 mL). The homogeneous mixture was stirred for 30 min, and the nanoparticle suspensions were transferred into dialysis tubing (Specra/Pro, 12–14 kDa MWCO, Fisher Scientific) followed by dialysis against DI water for 24 h. Nanoparticle size and polydispersity were analyzed by dynamic light scattering (DLS, Wyatt, DynaPro). Zeta potentials were determined by Zetasizer Nano Z (Malvern Instruments, UK), and data were analyzed using DTS Nano software.

### Nanoparticle stability

Nanoparticle stability was performed as previously described^27^. The stock suspensions of nanoparticles (1 mg/mL) were diluted (500 μg/mL) with regular neuronal medium (for cell labeling) and glucose (for slice labeling), respectively. Each sample (100 μL) was injected into a 96-well microtiter plate. Mineral oil was added on the top of each well via syringe to form a thin layer to prevent evaporation. The plate was put into the DLS instrument, protected from light, set to 37 °C, and the sizes and polydispersities of the nanoparticles were recorded over the course of 6 h. For optical stability of O_2_ NPs, the total emission was measured before and after incubation.

### Primary neuronal culture

Animals were handled according to a protocol approved by the University of Virginia Animal Care and Use Committee (ACUC), and efforts were made to minimize animal stress and discomfort. Cultures were prepared from postnatal day 0 to postnatal day 1 (P0–P1) C57BL/65 mice using methods described previously^37^. The newborn pups were decapitated, and their brains removed and placed in cold HEPES-buffered Hanks’ balanced salt solution (HEPES-HBSS). The hippocampi were removed under a dissecting microscope and collected in a small petri dish containing HEPES-HBSS. The hippocampi were transferred to 0.125% trypsin HEPES-HBSS and were incubated for 15 min at 37 °C. Trypsin solution was centrifuged for 7 min at 700 rpm, and the supernatant was replaced with 5 mL HEPES-HBSS. The cells were rinsed with a warm surgical medium by centrifuging for 7 min at 700 rpm and discarding the supernatant. Hippocampi were triturated until no fragments of tissue remained. Cell density was determined by trypan blue exclusion. Culture dishes were coated with poly-lysine and filled with 2 mL of surgical medium, which was prepared from Dulbecco’s modified Eagles medium (DMEM) and F-12 supplement (1:1) (Invitrogen) with 10% fetal bovine serum (heat-inactivated, Invitrogen), 2 mM L-glutamine (Invitrogen), and penicillin–streptomycin (100 U/mL). Cells were plated at a density of 50,000 per 35 mm^2^ dishes and kept in a 5% CO_2_ incubator at 37 °C. After 24 h, the surgical medium was changed to a serum-free neuronal regular medium containing DMEM and neurobasal (2.28:1) with 2% B27 and 2 mM glutamine. The medium was replaced with fresh regular medium every two days.

### Cell labeling

Primary neurons were 14 days in vitro (DIV) at the time of imaging for best observation of synapses. Blue NPs were diluted in a regular medium to yield a final concentration of 500 μg/mL. Cells were incubated in the above solution at 37 °C in a 5% CO_2_ incubator for 1 h. After staining, the cells were washed twice with HEPES-ACSF (pH 7.4, osmolarity 314–316 mOsm) containing (in mM) 147 NaCl, 2.6 KCl, 2 CaCl_2_, 3 MgCl_2_, 10 glucose, 10 HEPES and were kept in such medium during imaging. To label mitochondria, cells were incubated with a regular medium containing MitoTracker Red FM (250 nM) for an additional 30 min before washing with HEPES-ACSF.

### Brain slice preparation

Acutely isolated brain slices were prepared as previously described^38^. The animals were sacrificed under anesthesia, and the brains were removed and immersed immediately in ice-cold (4 °C) oxygenated (95% O_2_, 5% CO_2_) sucrose-ACSF (pH 7.4, osmolarity 300–310 mOsm) containing (in mM) 56.5 NaCl, 2 KCl, 5 MgSO_4_, 25 NaHCO_3_, 1 KH_2_PO_4_, 0.5 CaCl_2_, 10 glucose, 113 sucrose. Coronal hippocampal slices (200 μm) were prepared with a vibratome (VT1200S; Leica, Wetzlar, Germany) and were incubated in oxygenated sucrose-ACSF at 34 °C for 30 min before labeling or recording.

### Slice labeling

To minimize NP aggregation, O_2_ sensing NPs were suspended in 3 M glucose with NP final concentration at 500 μg/mL. Slices were labeled by incubating in the oxygenated NP glucose solution for 2 h. Slices were washed and kept in oxygenated sucrose-ACSF prior to imaging.

### Imaging

Imaging was performed using a Zeiss 780 confocal/multiphoton microscope system at the UVA Keck Imaging Center with Zeiss Zen software for image acquisition (Carl Zeiss, Oberkochen, Germany). For imaging cultured neurons, the excitation wavelengths used to visualize blue NPs and MitoTracker Red FM were 405 nm and 595 nm, respectively. Emission filters ranged from 410 to 500 nm for blue, and 600 nm to 700 nm for red. Magnifications of 40× or higher were used in regions of interest. For imaging live slices, the brain slices were anchored in a closed bath chamber and perfused with oxygenated sucrose-ACSF. A 405 nm laser was used as the excitation source, and emission ranged from 410 to 500 nm to capture fluorescence and 500 nm to 650 nm to capture phosphorescence. For high K^+^ measurements, the K^+^ concentration was increased from 2 to 5 mM in the perfusion solution (56.5 NaCl, 5 KCl, 5 MgSO_4_, 25 NaHCO_3_, 1 KH_2_PO_4_, 0.5 CaCl_2_, 10 glucose, 107 sucrose). Ratiometric imaging was processed in MATLAB by taking the ratio of fluorescence to phosphorescence.

### Electrophysiology

Electrophysiological properties of DGC neurons were recorded using a whole-cell patch-clamp technique as described previously^39^. Animals were aged around four weeks at the time of the study. The slices were perfused with oxygenated ACSF. Current-clamp recordings were performed for the measurement of cell membrane properties, and for filling biocytin into the cell. Recordings were performed in tight-seal (seal ≥ 1 Gigohm) current-clamp mode. The recording solution ACSF (in mM): 126 NaCl, 2.5 KCl, 26 NaHCO_3_, 2 CaCl_2_, 2 MgCl_2_, 1.25 NaH_2_PO_4_, 10 Glucose. The internal solution was (in mM): 135 K-gluconate, 7 KCl, 10 HEPES, 0.5 EGTA, 2.5 NaCl, 4 Mg-ATP, and 0.3 Na-GTP. Electrode capacitance was electronically compensated. Access resistance was continuously monitored, and if the series resistance increased by 20% at any time, the recording was terminated. Currents were filtered at 2 kHz, digitized using a Digidata 1322 digitizer (Molecular Devices, Sunnyvale, CA, USA), and acquired using Clampex 10.2 software (Molecular Devices). All recordings were performed at 30 °C. The currents were analyzed as described before using the MiniAnalysis software.

## Supplementary Information

Supplementary Figures.

## Data Availability

The datasets generated and analyzed in this work are available from the corresponding author upon reasonable request.
