# Supplementary material for: Difluoroboron β-diketonate polylactic acid oxygen nanosensors for intracellular neuronal imaging
Source: Sci Rep. 2021 Jan 13;11:1076. doi: 10.1038/s41598-020-80172-w (PMC7806623; doi:10.1038/s41598-020-80172-w)
Supplement: Supplementary file 1 — Supplementary Figures. [file 41598_2020_80172_MOESM1_ESM.docx]

**Supplementary Information for**

Difluoroboron β-Diketonate Polylactic Acid Oxygen Nanosensors for Intracellular Neuronal Imaging

Meng Zhuang^1^, Suchitra Joshi^2^, Huayu Sun^2^, Tamal Batabyal^2^ Cassandra L. Fraser*^1^, Jaideep Kapur*^2, 3, 4^

^1^Department of Chemistry, University of Virginia, Charlottesville, Virginia 22904, United States

^2^Department of Neurology, University of Virginia, Charlottesville, Virginia 22903, United States

^3^Department of Neuroscience, University of Virginia, Charlottesville, Virginia 22903, United States

^4^UVA Brain Institute, University of Virginia, Charlottesville, Virginia 22903, United States

*Co-corresponding Authors: [cf4n@virginia.edu](mailto:cf4n@virginia.edu), [jk8t@virginia.edu](mailto:jk8t@virginia.edu)


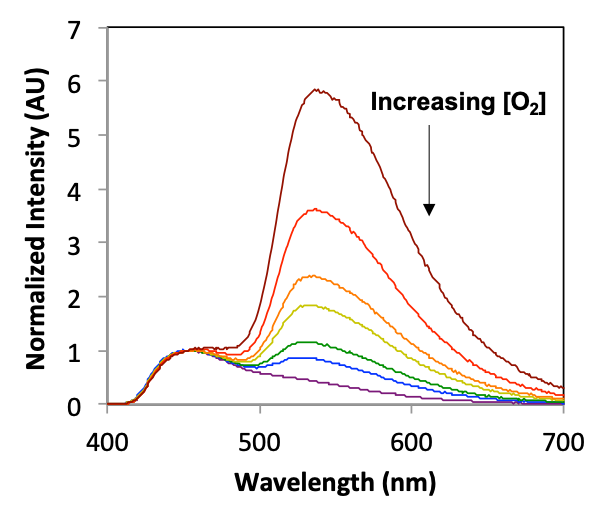


Figure S1. Total Emission of O_2_ sensing NPs at Varying Oxygen Levels. Oxygen concentration is from 0 to 21 %.

**
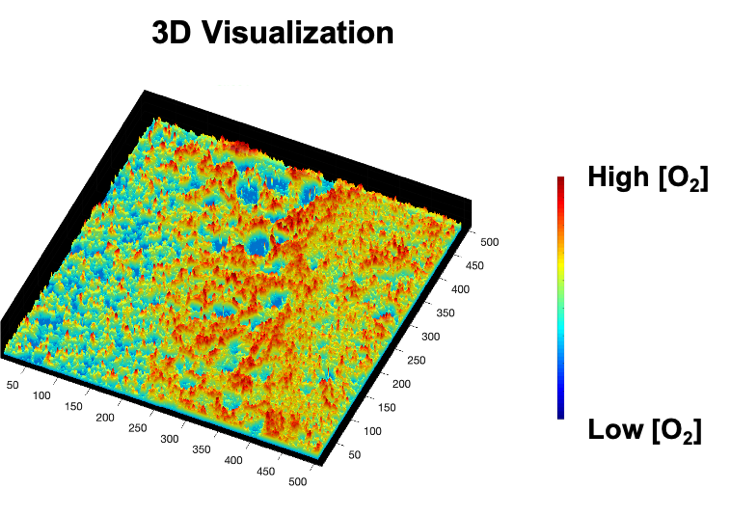
**

Figure S2. 3D Ratio Visualization. The image was processed from Figure 4B


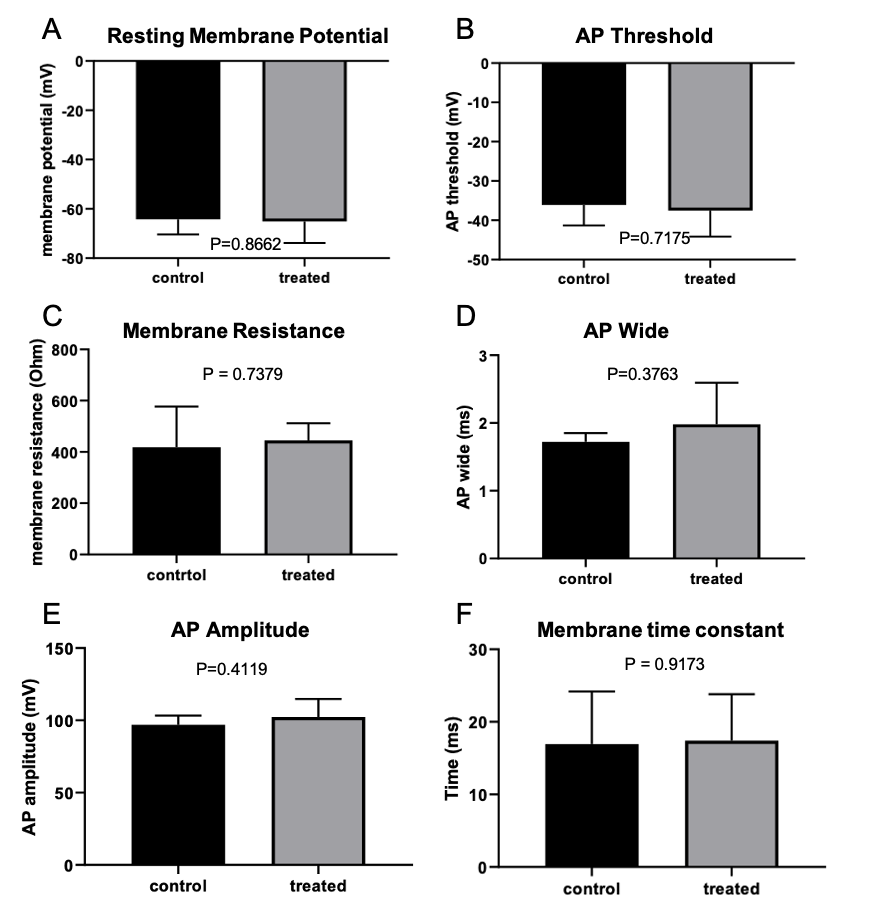


Figure S3. Electrophysiological Properties for Nanoparticle Treated Slice and Control.


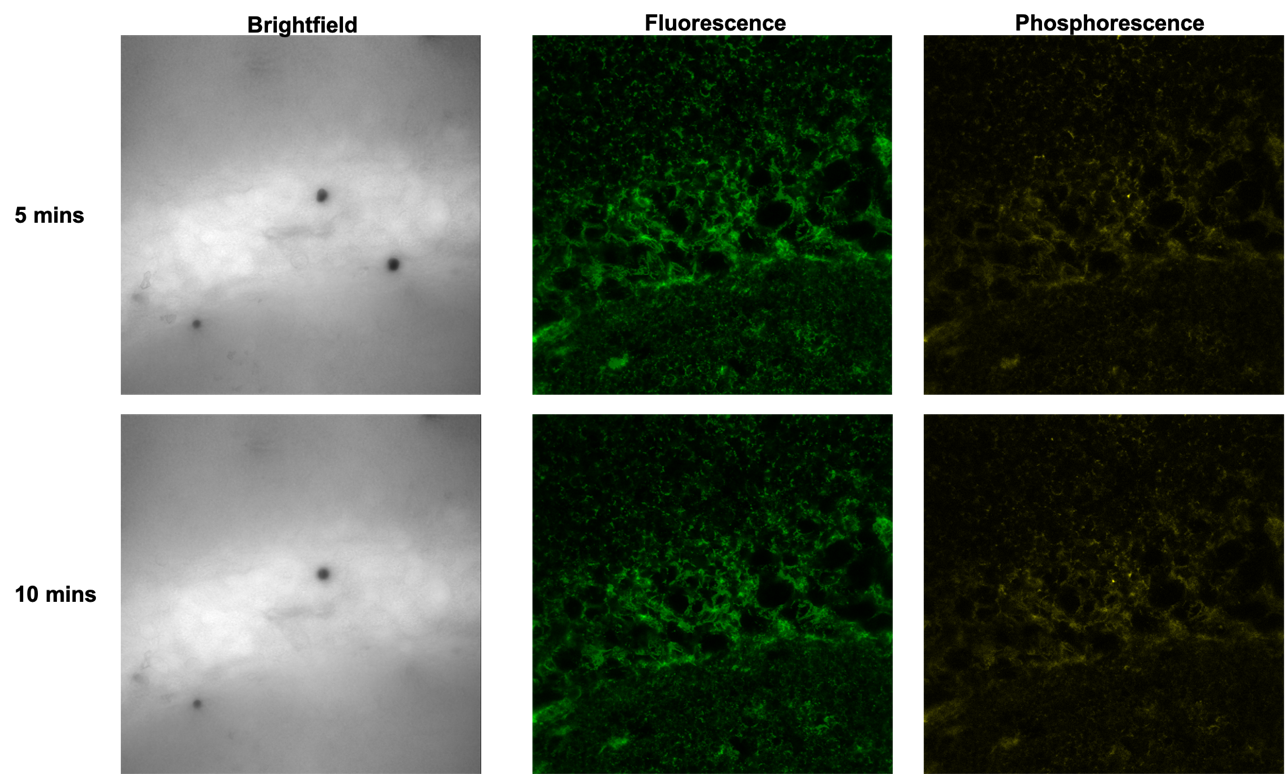


Figure S4. Raw Images of Slices after Oxygen Supply is off at 5 mins and 10 mins.
